# Walking Cadence to Exercise at Moderate Intensity for Adults: A Systematic Review

**DOI:** 10.1155/2017/4641203

**Published:** 2017-03-28

**Authors:** J. Slaght, M. Sénéchal, T. J. Hrubeniuk, A. Mayo, D. R. Bouchard

**Affiliations:** ^1^Faculty of Kinesiology and Recreation Management, University of Manitoba, Winnipeg, MB, Canada; ^2^Faculty of Kinesiology, University of New Brunswick, Fredericton, NB, Canada

## Abstract

*Background*. Most adults choose walking as a leisure activity. However, many do not reach the international physical activity guidelines for adults, which recommend moderate intensity aerobic activity for at least 150 minutes/week in bouts of 10 minutes.* Purpose*. This systematic review provides an update on the walking cadence required to reach moderate intensity in adults and older adults, identifies variables associated with reaching moderate intensity, and evaluates how walking cadence intensity should be measured, but the main purpose is to report the interventions that have been attempted to prescribe walking cadence to increase time spent at moderate intensity or other outcomes for adults and older adults.* Methods*. SportDISCUS, Scopus, and PubMed databases were searched. We identified 3,917 articles and 31 were retained for this systematic review. Only articles written in English were included.* Results*. In general, 100 steps/minute is prescribed for adults to achieve moderate intensity, but older adults may require a higher cadence. Currently, few studies have explored using walking cadence prescription as an intervention to increase physical activity levels.* Conclusion*. Prescribing walking cadence as a way to increase physical activity levels has potential as a practical and useful strategy, but more evidence is required to assess its ability to increase physical activity levels at moderate intensity.

## 1. Introduction

The World Health Organization recognizes physical inactivity as the fourth leading cause of death worldwide [1]. Although many adults are aware of the health benefits related to physical activity, many still fail to exercise regularly and do not meet the global physical activity recommendations [2–4]. Most national and international physical activity recommendations suggest all adults should reach moderate-to-vigorous intensity activity for at least 150 minutes/week in bouts of 10 minutes or more [2, 5, 6].

The reasons people fail to achieve such physical activity goals include the following: lack of knowledge regarding the physical activity guidelines [7], accessibility to facilities [8], lack of self-efficacy in regard to engaging in physical activity [9], and perceived lack of time [10]. Many health professionals prescribe walking as a way to reach the international or national guidelines [11, 12]. However, reaching the guidelines while walking may be confusing if an individual does not know how to achieve a minimum of moderate intensity, as suggested.

Walking is the most common method of exercise among adults [13]. One of the initial recommendations for walking for health was to achieve 10,000 steps/day, which was found to motivate inactive people to take more steps/day [14], but this recommendation neglected to include intensity and bout length requirements, which are critical components in order to reach the physical activity guidelines and health outcomes [14]. As a consequence, studies have shown that reaching 10,000 steps/day was not necessarily associated with expected outcomes [14].

Walking cadence (steps/minute) has been suggested as an approach that may be useful in assessing compliance to current physical activity guidelines, because although it still relies on a step count, it also places emphasis on the speed of the steps, therefore, acting as a method to estimate the intensity. Previous research has suggested that monitoring steps/minute in conjunction with the time spent at that cadence could be preferential to the current 10,000 steps/day guideline, as it promotes an increased intensity. In 2012, a systematic review aimed to present and summarize the potential for cadence to represent behavioural patterns of ambulatory activity in free-living adults [15]. The authors concluded that cadence could potentially be used effectively in different study designs or settings for exercise prescription. The current review focuses on literature surrounding the prescription of walking cadence in order to provide an update on the recommended cadence (steps/minute) to reach moderate intensity in adults and older adults. Moreover, this study will identify variables that influence the achievement of moderate intensity, evaluate optimal measurement techniques, and report on the findings of interventions that have prescribed walking cadence for adults and older adults in an attempt to increase meaningful exercise levels.

## 2. Methods

A comprehensive literature review was employed to identify relevant articles on this topic. The following databases were searched: SportDISCUS (1975–April 15, 2016), Scopus (coverage varies according to journal publications), and PubMed (1964–April 15, 2016). An initial strategy was created for SportDISCUS and then adapted to suit the two additional databases. Limits were applied to year (2000–2016) and, in the case of SportDISCUS, peer-reviewed journals. The search strategies are presented in the Appendix. In total, 3,972 records were identified through the initial database search and imported into the RefWorks citation management database. Here, duplicates were removed, resulting in 3,571 records for screening. The remaining records were then assessed according to predetermined inclusion criteria. The criteria included the following: adults aged 19 or more, not being about a specific clinical population, investigating specifically walking cadence (e.g., steps/minute, stepping rate) for validity purposes, or an intervention aiming to test the benefits of walking at one or different walking cadences. Based on these criteria, results were narrowed further, creating a list of publications suitable to the theme of walking cadence. A total of 168 articles were identified and included in the abstract review process. Two authors individually reviewed the abstracts and, following deliberation, agreed upon 31 articles for inclusion in the systematic review (Table 1).

## 3. Results

### 3.1. Walking Cadence Required to Reach Moderate Intensity in Adults

In 2012, a review was published by Tudor-Locke and Rowe that attempted to identify the required cadence to reach moderate intensity [15]. The authors outlined five studies that evaluated the cadence necessary to reach moderate intensity, all of which were completed in controlled clinical settings [16–20], with adults ranging in age from 20 to 60 years. These tests consisted of participants walking at multiple speeds on a treadmill, with intensity evaluated based on reaching 3 metabolic equivalents (METs) [16, 18–20]. Each study acknowledged the implicit variation among individuals, but they all concluded that a prescription of 100 steps/minute was sufficient when trying to attain moderate intensity, when defined as achieving 3 METs [16–20]. When using 3 METs as the threshold for moderate intensity, one study concluded that only 45% of adults reached moderate intensity [18], suggesting that individual walking cadence prescription is needed when it comes to intervention to increase time spent at moderate intensity when walking.

Fewer studies have been completed involving older adults attempting to determine the walking cadence necessary to reach moderate intensity. This is problematic, as previous research has displayed that older adults have different gait patterns when compared to a younger population [21]. Recent research completed by our group [22], along with others [23, 24], has suggested that older adults need to walk at a cadence greater than 100 steps/minute to achieve moderate intensity. Despite changes in gait patterns that occur with age, studies have shown that older adults typically walk at a faster cadence than their younger counterparts when walking at a self-selected pace within a clinical setting [16–20, 22–24]. However, these cadence rates are not being achieved outside of clinical assessments in daily living [24, 25], which, thus, likely prevents achieving the desired health benefits. For example, Dall and colleagues observed a sample of 117 individuals ranging from 30 to 62 years of age and found an average free-living cadence of 76 ± 6 steps/minute [26]. Similarly, when you look at an even larger sample of adults represented by the National Health and Nutrition Examination Survey (NHANES) [25], a cadence equal to or greater than 100 steps/minute becomes a rarity in the typical adult population [25]. These findings represent a reduced cadence when compared to evidence observed in the older adult population provided by Tudor-Locke and colleagues. In their study, participants between 61 and 81 years of age averaged a walking cadence of 104 steps/minute (men) and 111 steps/minute (women) when asked to walk at their self-selected speed in a clinical testing environment. Furthermore, this research identified that, despite the capability of older adults of achieving such a high cadence, it is scarcely achieved in free-living scenarios, with older adults spending less than 10 minutes/day above the “normal cadence” walked in the clinic [24].

Finding methods to adequately implement the use of a prescribed cadence to reach moderate intensity while walking outside of a clinical setting should be the focus of future research. The health benefits of achieving such targets were underlined by Brown and colleagues in 2014, who found that walking at a cadence > 100 steps/minute was associated with a 21% reduction in all-cause mortality. More important, each increase of ten steps/minute resulted in an additional 4% reduction in all-cause mortality [27]. However, an often overlooked portion of physical activity recommendations is the requirement for individuals to complete the activity in bouts of 10 minutes or more. Therefore, it is valuable to ensure that walking at a rate greater than 100 steps/minute equates to achieving moderate intensity physical activity for a continuous 10-minute bout. In a study with a focus on free-living conditions, Ayabe et al. found that accumulating 100 steps/minute by completing 1,000 steps in 10 minutes, or 3,000 steps in 30 minutes, may not be enough to achieve moderate intensity [28]. Participants wore an accelerometer for seven consecutive days to measure typical physical activity levels, and although results showed 20 of the 33 participants averaged 30 minutes/day in moderate-to-vigorous physical activity, this was not accumulated in 10-minute bouts. This study [28], along with that completed by Tudor-Locke et al. [24], shows that achieving a cadence of 100 steps/minute does not necessarily translate into successfully walking at moderate intensity for 10-minute bouts. Therefore, it remains important to develop and identify strategies that not only assist adults in reaching the necessary walking cadence but will also enable them to maintain it for at least 10 minutes to achieve the physical activity recommendations and elicit optimal health benefits [2].

### 3.2. Variables Associated with Reaching Moderate Intensity

Research has shown that there are a few key variables that can significantly impact the walking cadence prescription to reach moderate intensity. It has been found that an individual's height plays a significant role in the walking cadence necessary to achieve moderate intensity [19], especially in adults. The study, completed by Rowe et al., was performed on 75 adults with a mean age of 32.9 ± 12.4 years at three different speeds (slow, medium, and fast) on a treadmill and on an overground walking trail [19]. The study included participants with heights ranging from 60 to 78 inches (152–198 centimetres), and the subsequent walking cadence to reach moderate intensity based on 3 METs ranged from 90 to 113 steps/minute [19]. The authors were able to show that as height increases, the walking cadence needed to reach 3 METs decreases [19]. As such, although 100 steps/minute is the average cadence prescription, the height of an individual should be considered when trying to select an appropriate walking cadence to reach moderate intensity [19, 23], especially in a clinical setting where the exercise prescription needs to be specific to each person. Two studies reported that leg length was also associated with the walking cadence required to reach moderate intensity [19]. However, it was found that height and leg length had a strong correlation coefficient of 0.90 [17]. While either measure may be utilizable, Rowe et al. [19] argue that height would be easier to measure than leg length for prescribing walking cadence. Finally, age was not identified as a predictor for the walking cadence to reach moderate intensity, despite studies reporting that the walking cadence required for older adults to reach moderate intensity is greater than 100 steps/minute [23, 29] and older adults naturally choosing a greater walking cadence (men:108 to 114; women: 109 to 119) [21].

A final factor that may have an effect on walking cadence is body weight or body mass index (BMI). One study has shown that men of a healthy weight aged between 30 and 47 years were more likely to have a higher walking cadence than overweight men when walking over a 15-metre distance (obese: 103 ± 8 steps/minute; nonobese: 116 ± 13 steps/minute) [30]. In 2011, Ayabe et al. [31] compared activity levels in normal weight participants (BMI < 25 kg/m^2^) to overweight and obese participants (BMI > 25 kg/m^2^). Intensity was measured based on cadence (light < 100 steps/minute, moderate 100–129 steps/minute, and vigorous > 130 steps/minute). The results showed that total number of steps and time spent in light, moderate, and vigorous intensity were significantly less in the overweight group compared to the normal weight group. Furthermore, the total time spent at moderate intensity per day in the overweight group averaged 40% less than that of the normal weight group [31]. A recent study from our group has also reported that the walking cadence required to reach moderate intensity was reduced by 2.5 steps/min for each 10 kg increase in body weight [22].

Although these factors are associated with walking cadence, it is important to note that using these elements to predict the cadence needed to reach moderate intensity may not be accurate. For example, Serrano et al. reported that, after observing the capabilities of these variables (i.e., height, weight, leg length, and age) to predict walking cadence, the best coefficient of variation was 13%, showing widespread variation and inaccuracy in making predictions [22]. As a result, some key characteristics need to be considered in order to properly individualize walking cadence and ensure that people reach at least moderate intensity while walking. However, a gap remains in the ability of these to predict the individualized walking cadence rates necessary to reach moderate intensity. This is likely due to a lack of adequately researched variables that could be influencing our ability to predict cadence. There is the possibility that alternative, biomechanically focused variables such as sway/balance, plantar pressure parameters, stance time, and centre of balance estimations are significantly influencing the ability of individuals to walk at the cadence necessary to achieve moderate intensity. Subsequently, further research investigating these variables and their influence on predicting cadence is needed.

### 3.3. Strategies, Tools, and Interventions

To date, three strategies have been explored to encourage individuals to walk at moderate intensity with a walking cadence prescription, mostly in a cross-sectional manner: first, through the utilization of auditory cues, using metronomes or music for pacing in apparently healthy adults [32–35] and clinical populations [36, 37]; second, by asking participants to self-perceive intensity [32, 38, 39]; third, by providing a pedometer with a target cadence to follow [40–42]. As of now, few pedometers, displaying walking cadence at different intensities, have been validated [34, 46].

#### 3.3.1. Auditory Cues

Rowe et al. [32] conducted a study to evaluate the efficacy of rhythmic auditory stimuli in regulating moderate intensity walking cadence in a laboratory setting. Participants completed a treadmill test at 2.7 mph, based on ACSM guidelines for achieving moderate intensity [47], and their cadence was recorded. Next, they were asked to walk on a flat track following a metronome that was set to mimic the cadence they achieved during the treadmill test, which on average was 114 ± 8 steps/minute, and results showed that participants were able to maintain the set pace [32]. Another study testing older adults has shown that music and metronome do not offer the same ability to follow pace, with music being better [36]. These findings support the hypothesis that people are responsive to the use of auditory feedback for walking cadence. As such, this could be a strategy worth exploring in future interventions. To date, no interventions have tested if using auditory cues led to an increase in physical activity level at moderate intensity. However, a study involving 12 college students found that greater music tempo led to more intensity and found an association between music speed and exercise intensity while cycling for 25 minutes [43].

#### 3.3.2. Self-Perceived Intensity

In 2014, Pillay and colleagues explored whether simply instructing people to walk at a self-selected “brisk pace” was adequate for achieving moderate intensity in adults [33]. The average self-selected brisk walking pace was found to be 118 ± 9 steps/minute, similar to the predicted cadence for reaching moderate intensity of 122 ± 37 after adjusting for sex, age, and aerobic fitness level [33]. Another common self-perception strategy is the “talk test,” a subjective method used to estimate cardiorespiratory exercise intensity while walking. The talk test aims to estimate moderate intensity with the assumption that it has been achieved when an individual can no longer hold a comfortable conversation when performing an activity. It is assumed that, at this point, the individual has achieved ventilatory threshold [39]. The talking test was previously validated as capable of accurately identifying moderate walking intensity alongside heart rate, oxygen consumption, and ventilatory threshold [38]. Although these strategies have the advantage of affordability, as neither requires any equipment, objective measures remain the preferred method to easily confirm a prescription as being effective [44].

#### 3.3.3. Pedometers

Two studies have focused on the utility of pedometers to measure walking cadence [41, 42]; one randomized control trail has been registered [45], and one is in press at the Journal of Aging and Physical Activity [40]. Marshall et al. [41] recruited 180 females aged 18 to 55 years and compared three groups with different daily step goals: (i) self-selected steps goal (control); (ii) 10,000 steps; (iii) 3,000 steps in 30 minutes. Aside from the different approaches, all three groups received the same 12-week theory-based physical activity intervention. Among the three groups, the group that was prescribed 3,000 steps in 30 minutes, which translates into a walking cadence of 100 steps/minute, had the greatest increase in moderate-to-vigorous time spent in 10-minute bouts [41]. Participants in both intervention groups (10,000 steps/day and 3,000 steps in 30 minutes) spent significantly more time in moderate-to-vigorous intensity compared to the control group, but only the walking cadence prescription group (3,000 steps in 30 minutes) significantly increased time in moderate-to-vigorous intensity in bouts of at least 10 minutes [41], as outlined in national and international physical activity guidelines [5, 48].

Two more studies have recently been published. A study by Slaght et al. [40] tested the ability for older adults to increase the time spent at moderate intensity in 10-minute bouts. The intervention was divided into two phases, each lasting six weeks. During the first six-week phase of the study, all participants were asked to walk a minimum of 150 minutes/week at no specific intensity. For phase two, all participants aimed to walk at least 150 minutes/week, with the instruction of doing this in 10-minute bouts at moderate-to-vigorous intensity. Participants in one group received an individualized walking cadence prescription and a pedometer (StepsCount, StepRx, Ontario, Canada) that provided visual feedback indicating whether their walking cadence was achieving moderate-to-vigorous intensity in 10-minute bouts. During phase 2 only the intervention group increased time at moderate intensity and moderate intensity in 10-minute bouts compared with phase one and the control group.

In another study, a pedometer (Omron HJ-151) was used in addition to a lifestyle modification program as part of a 12-week intervention for weight loss in 90 overweight/obese adults between the ages of 35 and 64 years [42]. Participants were randomized into two groups: lifestyle + pedometer or lifestyle only. The results show that participants receiving the pedometer did not increase the total steps/day but accumulated significantly more steps after intervention with greater walking cadence. For example, the participants in the pedometer group increased step counts within the 100–119 range (463 ± 1092 versus 56 ± 546 step counts; *p* = 0.01). Finally, a randomized control trial is currently underway aiming to compare the ability of three one-month pedometer-based walking interventions and their impact on the blood pressure of sedentary/low activity level postmenopausal women, a population at risk of cardiovascular disease. The three groups are similar to those found in Marshall et al. [41]: (i) 10,000 steps/day (with no guidance on walking intensity); (ii) 10,000 steps/day and at least 30 minutes in moderate intensity (>100 steps/min); and (iii) a control group [45]. This study will provide valuable, high level evidence in identifying the effectiveness of providing direction on walking cadence intensity to exercise prescription models. Furthermore, it will greatly enhance our knowledge on the effectiveness of a walking cadence prescription to improve health outcomes, with a specific focus on blood pressure in connection with cardiovascular health [45].

These interventions are the first steps towards transferring the current knowledge regarding walking prescription into real-world settings and increasing the number of adults reaching moderate intensity while walking. More interventions need to be completed to determine the optimal and most sustainable methods for assisting the public towards reaching moderate intensity in bouts of at least 10 minutes. Once more research is completed in this field, a meta-analysis can be conducted to quantify the average increase in time spent in moderate intensity when using walking cadence as a strategy to eventually meet the national and international physical activity guidelines. Future studies should also include a long-term follow-up to see if the interventions lead to long-term changes in physical activity behaviour, along with health indicators.

## 4. Conclusion

The amount of research supporting walking cadence as a means for measuring and achieving moderate intensity has grown substantially over the past ten years. Based on the literature using a threshold of 3 METs as a measure of achieving moderate intensity, the general prescription of 100 steps/minute is sufficient for most of the adult population to reach moderate intensity [16, 18–20]. With that stated, older adults might require a higher walking cadence prescription to reach that same intensity [23, 24]. Certain characteristics can dramatically impact the walking cadence prescription to reach moderate intensity. Strategies to assist people in achieving moderate intensity through walking cadence have started to grow, with some evidence supporting the use of auditory cues or pedometers [32–34]. Very few studies have explored using a walking cadence intervention to get the inactive adult population more active [40–42, 45]. Since walking is constantly reported as being the most popular choice of activity among adults of all ages [13], the notion of using walking cadence to achieve the optimal intensity related to health outcomes needs to be further investigated and transferred to different community groups that promote walking for exercise. In conclusion, although walking cadence might be a practical and useful tool to prescribe walking intensity, more research is needed to demonstrate the long-term benefits of this strategy to increase the time people walk at moderate-to-vigorous intensity.

## Figures and Tables

**Table 1:** Description of studies included in the systematic review.

Authors, year, reference #	Purpose	*N*	Age (years)	Walking cadence to reach moderate intensity	Variables associated with reaching moderate intensity	Strategies, tools, and interventions	Main outcomes
Tudor-Locke and Rowe (2012) [15]	Summarize the potential for using cadence to measure and promote intensity of activity	Review article	—	Most studies find that 100 s/m elicits 3 METs (mod intensity)	Focuses on what we can do with cadence	Cadence appears to be sensitive to change with intervention	100 s/m = 3 METS Pedestrian cadence in natural conditions = 115 s/m. Lower cadence in free living

Abel et al. (2011) [16]	Identify step rate thresholds for different intensities	19	28 ± 7	Treadmill test at six standardized speeds. O2 consumption versus step rate	—	—	Men: mod = 94 vig = 125 Women: mod = 99 vig = 135 100 s/m = practical public health recommendation for mod intensity

Beets et al. (2010) [17]	Examine impact of leg length on steps per day associated with MPA	20	26 ± 5	Overground walking, 5 speeds, portable gas analyser	Leg length (related to step frequency)	—	100 s/m = 3 METs Increase leg length = decrease (−1.15) s/m Range for MPA s/m = 85–111

Marshall et al. (2009) [18]	Creating a pedometer-based guideline for PA recommendations	97	32 ± 11	4 speeds, O2 measured and steps. Step rate cut-point for mod = 3 METS	—	—	Men 3 METs = 92–102 s/m Women 3 METs = 91–115 s/m

Rowe et al. (2011) [19]	What is the self-selected “brisk” walking pace in inactive adults	25	34 ± 13	Mod intensity treadmill walking trial, steady state 02 measured	Self-selected brisk walk = higher cadence than mod pace	Used metronome to assist in pacing mod intensity	Self-selected brisk = 124 ± 8 s/m. Mod pace (metronome) = 114 ± 8 s/m with EE > 3 METS

Tudor-Locke et al. (2005) [20]	Pedometer-determined step count guidelines for walking intensity	50	18–39	6 min exercise bouts at 3 treadmill speeds and steady state VO2 recorded	—	—	Pedometer cut-points for minimal mod intensity = 96 s/m (men) and 107 s/m (women)

Nagasaki et al. (1996) [21]	Walking patterns and finger rhythm	1134	≥65	—	Step ratio = step length/cadence Age Finger festination (rhythmic movement)	—	Older = shorter steps Increased finger festination = increased step rate (smaller walk ratio)

Serrano et al. (2016) [22]	Evaluate walking cadence needed to reach moderate intensity in older adults & develop an algorithm for prescription	121	69 ± 8	Moderate intensity = when participants reached 40% of peak oxygen consumption on an indoor flat surface	Body weight, stride length, and height	Created an algorithm to predict mod intensity walking cadence	Mean walking cadence to reach mod intensity was 115 ± 10 s/m

Peacock et al. (2014) [23]	Stride rate and walking intensity in healthy older adults	29		3 ground and 3 treadmill trails (slow, medium, and fast) VO2 measured (indirect calorimetry)	Stride rate, age, and height have a significant effect (*p* < 0.01) on walking intensity	Synchronization between stride rate and music tempo found that music can be a useful way to guide walking cadence	Mean s/m exceeded minimum thresholds MVPA; slow (111 ± 12 s/m), medium (118 ± 11 s/m), and fast (124 ± 11 s/m) instructions

Tudor-Locke et al. (2013) [24]	Compare clinical and free-living cadence in older adults	15	61–81	Gait speed (cm/sec) & cadence measured for 1 week	Steps/day, normal versus dual task walking, pedometer versus accel steps/day	This group was able to walk at cadences > 100 s/m, but, on average, <10 min/day was spent above this cadence	Shown that these adults were capable of walking at cadences ≥ 100 s/m, but this was uncommon in daily life

Tudor-Locke et al. (2012) [25]	Analysis of NHANES for peak 30 min and 1 min cadence	3522	20+	Peak 30-minute cadence (highest s/m in a day, not necessarily consecutive minutes) & peak 1-minute cadence (the highest single minute in a day)	Sex, age, and BMI	—	30 min peak = 71 s/m 1 min peak = 100 s/m Both show decline in cadence with age and obesity

Dall et al. (2013) [26]	Comparing rate of stepping and number of steps in a minute epoch	117	46 ± 16	—	Comparing step accumulation versus cadence	—	Most walking was not done continuously so cadence cannot be determined from step accumulation alone. These 2 measures are not interchangeable

Brown et al. (2014) [27]	Determine if ability to walk ≥100 s/m predicts mortality in older adults	5000	70.6	Cadence calculated using 2.4 m walk and separated into ≥100 s/m versus <100 s/m	Cadences effect on mortality	—	Ability to walk ≥100 s/m predicted a 21% reduction in all-cause mortality. Each 10-step increase predicted a 4% reduction

Ayabe et al. (2013) [28]	Relationship between 1000 steps in 10 min (1k) and 3000 in 30 min (3k)	33	53 ± 19	Accelerometer measured light, mod, and vigorous intensity	Bouts of exercise intensity >10 minutes	—	# of steps (at all intensities) was higher on 1k or 3k days 1k duration not correlated with MVPA in >10 min bouts, unclear if cadence can be used to define MVPA

Taylor et al. (2010) [29]	Objective and subjective assessment of normal walking pace versus moderate intensity	10	54 ± 8	Normal walking pace measured using GPS over a 1 km outdoor walk and a timed 150 m trial	Height was significantly correlated with walking pace	Use of GPS for measurement has potential, allowing measurement in a real life setting	All participants walked at a pace considered as moderate intensity (≥1.34 m/sec)

Spyropoulos et al. (1991) [30]	Identify and compare components of walking gait between obese and nonobese men	12	30–47	Walking gait measured with cinematography in min/sec	Body weight	—	Obese = slower walking speed, shorter stride length, smaller cadence, and larger step width

Ayabe et al. (2011) [31]	Comparing stepping rate between normal weight and overweight/obese individuals	40	58 ± 8	Pedometer for 7 days to determine the number of steps and time spent in PA at <100, 100–129, 130 s/m	BMI (<25 kg m−2 or 25 kg m−2)	—	Overweight = sig. less steps/day, lower average stepping rate, and sig. shorter time in PA at 100 s/m

Rowe et al. (2013) [32]	Determine the role of height and stride length in mod intensity walking cadence	75	33 ± 12	3 overground and 3 treadmill trails (slow, medium, and fast) VO2 measured (indirect calorimetry)	Measured and 5 stride length variables and height	Height needs to be taken into consideration for more precise prescription of walking cadence	Height can cause cadence at mod intensity to vary more than 20 s/m (90–113)

Pillay et al. (2014) [33]	Examining the relationship between intensity and fitness/health outcomes for pedometer	70	32 ± 8	Pedometer classified aerobic as ≥60 s/m and nonaerobic as <60. Also collected total steps per day	Estimated VO2 max, BP, BMI, BF%, WC, age, gender, and total steps/day	—	Total steps/day and time accumulating “aerobic” steps inversely associated with BF%, BMI, WC, and systolic BP

Nielson et al. (2011) [34]	Determine accuracy of steps counts and EE as estimated	100	>50	Step counts and energy expenditure were estimated with a pedometer while walking 80, 90, 100, 110, and 120 s/m	—	Used metronome to help guide the 5 different step frequencies on the treadmill	Pedometer underestimated EE at 80 s/m and overestimated it at 90, 100, 110, and 120 s/m

Wittwer et al. (2013) [35]	Music and metronome cues effects on gait in healthy older adults	19	>65	—	Music versus metronome	Music = increase in gait velocity Both music and metronome = small increase in cadence (1 s/m)	Music and metronome cues produce different effects on gait spatiotemporal measures but not gait variability in healthy older adults

Wittwer et al. (2013) [36]	Effect of Rhythmic Auditory Cueing (RAC) on gait in people with Alzheimer Disease	30	80 ± 6	—	Music versus metronome	Both music and metronome = decreased stride length	RAC produced damaging effects on gait in a single session in this group with AD

Nascimento et al. (2015) [37]	Walking training on patients after stroke: a systematic review	211	—	—	—	Walking training with cueing of cadence	Walking training with cueing of cadence improves walking speed and stride length after stroke more than walking training alone

Foster et al. (2008) [38]	The talk test as a marker of exercise training intensity						Relationship between VT and the TT during various interventions and this suggests that the TT is suitable for exercise prescription

Persinger et al. (2004) [39]	Consistency of the talk test for exercise prescription	16	24				Support the hypothesis that the TT approximates VT on both treadmill and cycle

Slaght et al. (2016) [40]	Walking cadence prescription to reach the global physical activity recommendations in older adults	55	70	Mod intensity = when participants reached 40% of peak oxygen consumption on an indoor flat surface	Feedback on reaching mod intensity versus no feedback	Individualized cadence to reach mod intensity and feedback on ability to reach intensity = effective	Previously inactive older adults can increase time at mod intensity in 10-minute bouts weekly by using individually prescribed walking cadence

Marshall et al. (2013) [41]	Using step cadence goals to increase moderate-to-vigorous intensity physical activity	180	18–55	Accelerometer-based PA was measured at baseline and after 12 weeks	—	12-week intervention, 3 groups (self-selected, 10,000 steps per day, and 3000 steps in 30 min)	Group assigned 3000 steps in 30 min had more MVPA in ≥10 min bouts

Barreira et al. (2016) [42]	Pattern changes in step count accumulation and peak cadence due to a physical activity intervention	121	35–64	Accelerometer to measure different cadence bands & peak 1 min, 30 min, and 60 min cadence	—	2 groups; (1) diet and behaviour change, (2) diet and PA—increase steps/day and steps at MVPA	From the period before to the period after intervention no difference between groups for steps/day. Diet and PA group did accumulate significantly more steps at higher cadences

Waterhouse et al. (2010) [43]	Effects of music tempo upon submaximal cycling performance	12		Music tempo for cycling cadence	—	Faster tempo resulted in increased distance covered and cadence	Faster music = voluntarily increased workload

Bouchard et al. (2013) [44]	Can older inactive adults learn how to reach the required intensity of physical activity guideline?	25	≥65	2 groups used ≥40% HRR for moderate intensity; pedometer group used 100 s/m	Tool used to measure moderate intensity and ability to identify moderate intensity	3 different intervention methods (manual HR, HR monitor, and pedometer)	No group improved time in MVPA and HR monitor and pedometer groups increased total activity time and the pedometer group, though not significant, showed a tendency to be able to better identify mod intensity

Tudor-Locke et al. (2014) [45]	A randomized controlled trial of pedometer-based interventions differing on intensity messages	120	45–74	—	Comparing total steps versus cadence to elicit moderate intensity	3 groups; (1) 10,000 steps/day, (2) 10,000 steps/day & 30 min at mod intensity, and (3) control	This study will improve understanding on benefits walking volume and/or intensity have

s/m: steps per minute, MET: metabolic equivalent, O2: oxygen, mod: moderate, vig: vigorous, MPA: moderate physical activity, PA: physical activity, EE: energy expenditure, VO2: oxygen uptake, accel: accelerometer, NHANES: National Health and Nutrition Examination Survey, BMI: body mass index, GPS: Global Positioning System, sig: significantly, BP: blood pressure, BF%: body fat percent, WC: waist circumference, RAC: rhythmic auditory cueing, AD: Alzheimer's Disease, TT: talk test, VT: ventilatory threshold, HRR: heart rate at rest, HR: heart rate, and MVPA: moderate-to-vigorous physical activity.

## Appendix

### Electronic Search Strategies

- Sport Discus Search Strategy (retrieved 2,359 results from scholarly (peer-reviewed) journals when search tested 3rd week of April 2016)
- Scopus Search Strategy (retrieved 1,312 results when search tested 3rd week of April 2016)
- PubMed Search Strategy (retrieved 345 results retrieved when search tested 3rd week of April 2016)

*Concept 1 Walking Movement*

1. Step OR steps OR stepped OR stepping
2. 10,000 OR rate OR count OR goal
3. (1) AND (2)
4. Gait
5. Analysis
6. (4) AND (5)
7. Walk^*∗*^
8. Speed^*∗*^ OR pattern^*∗*^ OR pace^*∗*^ OR intensity
9. (7) AND (8)
10. “stride rate” OR pedometer^*∗*^ OR cadence
11. Combine Sets
12. OR/(3), (6), (9), (10)

*Concept 2 Adults (Population)*

(12) KEY (adult^*∗*^)
(13) TITLE (age^*∗*^ OR aging^*∗*^ OR adults^*∗*^)
(14) (12) OR (13)

*Concept 2 Assessment*

(15) Increas^*∗*^ OR exercise OR recommendation^*∗*^ OR assess^*∗*^ OR comparison^*∗*^ OR measur^*∗*^ OR “physical activity” OR accura^*∗*^

*Concept 3 Terms to Filter Out*

(16) Pregnan^*∗*^ OR child^*∗*^ OR cycl^*∗*^ OR danc^*∗*^
  Combine Sets
(17) (11) AND (12) AND (13) NOT (14)
